# Draft genome sequence of *Enterococcus gallinarum* strain TY-1 isolated from human saliva

**DOI:** 10.1128/mra.01045-25

**Published:** 2025-11-24

**Authors:** Qingli Zhang, Zichu Zhao, Nan Li, Qu Chen, Hailong Li, Jingjing Kong, Yan Zhang, Haili Zhang, Tingting Tang

**Affiliations:** 1 Department of Medicine, Qingdao Binhai University 563650, Qingdao, Shandong, China; 2 Department of Rehabilitation, The Affiliated Hospital of Qingdao Binhai University 832297, Qingdao, Shandong, China; Nanchang University, Nanchang, Jiangxi, China

**Keywords:** *Enterococcus gallinarum*, draft genome sequence, human saliva

## Abstract

We report the draft genome sequence of *Enterococcus gallinarum* strain TY-1 isolated from human saliva in China. The genome is 3.4 Mb in size with a G + C content of 40.5 % and encodes 3,150 predicted proteins, seven rRNAs, 56 tRNAs, four ncRNAs, and 76 pseudogenes.

## ANNOUNCEMENT

The genus *Enterococcus* renowned for its strong adaptability is commonly found in food, the environment, and the gastrointestinal tracts of humans and animals (1). *Enterococcus gallinarum* (*E. gallinarum*) is an underdiagnosed but clinically significant opportunistic pathogen (2); however, its isolation from human saliva and the genomic characterization of oral isolates remain limited. To address this gap, we isolated *E. gallinarum* strain TY-1 from human saliva.

For sample collection, participants were instructed to abstain from food and drink for 1 h prior to sampling and to expectorate 1–3 mL of naturally secreted saliva into a sterile collection tube. In a biosafety cabinet, 1 mL of each saliva sample was serially diluted with sterile normal saline to 10⁻³ to 10⁻⁵. Subsequently, 0.1 mL aliquots of each dilution were spread-plated onto MRS agar (Solarbio, China) supplemented with 0.5% (w/v) CaCO₃ (Solarbio, China). Inoculated plates were incubated anaerobically at 37°C for 48–72 h. Colonies exhibiting a clear CaCO₃-dissolution halo were selected, restreaked twice to ensure purity, and Gram-stained for preliminary identification as lactic acid bacteria. Genomic DNA was extracted from pure cultures using the TIANamp Bacteria DNA Kit (DP302; Tiangen, China). The 16S rRNA gene was amplified using universal primers 27F and 1429R (Table 1) and sequenced by Sanger sequencing for species identification.

**TABLE 1:** 16S rRNA gene and genome assembly statistics of *E. gallinarum* TY-1

Category	Specific content
16S rRNA gene	
GenBank accession	PX452890
Forward primer F(27) (5′–3′)	AGAGTTTGATCCTGGCTCAG
Reverse primer R(1492) (5′–3′)	CGGCTACCTTGTTACGACTT
Reaction mixture (25 µL)	1 µL DNA template (10–100 ng), 0.8 µL each primer (10 µmol/L), 12.5 µL 2× PrimeSTAR HS DNA polymerase (Cat. R010A, Takara, China), and 9.9 µL ddH₂O
Amplification condition	95°C 5 min and 32 cycles of 95°C 30 s, 55°C 45 s, and 72°C 90 s , 72°C 10 min
Sequencing kit and system	BigDye Terminator v1.1 (4336701, Thermo Fisher) and ABI3730XL (Applied Biosystems, USA)
Genome feature	
GenBank assembly	ASM5060942v1
Total length (bp)	3,363,870 bp
Number of scaffolds	52
Scaffold N50 kb	208.6
Scaffold L50	5
GC content (%)	40.5
Total genes	3,293
CDSs (total)	3,226
CDSs (protein-coding sequences)	3,150
rRNAs (5S, 16S, 23S)	4, 1, 2
Complete rRNAs (5S, 16S)	1, 1
Partial rRNAs (5S, 23S)	3, 2
tRNAs	56
ncRNAs	4
Pseudo genes (total)	76

A DNA library was prepared with the MagPure Bacterial DNA Kit (D6361-02; Magen, China). DNA was fragmented to 200–400 bp, end-repaired, 3′ .-adenylated, adapter-ligated, PCR-amplified, and size-selected with Hieff NGS DNA selection beads (Cat#12601) according to the manufacturer’s instructions. Libraries were quantified using a Qubit 4.0 fluorometer (Q33226; Thermo Fisher Scientific; default parameters were used, except where otherwise noted) using the high-sensitivity kit, size-verified on a 2% agarose gel, and paired-end sequenced (2 × 150 bp) on an Illumina NovaSeq 6000 (Sangon Biotech, China) (3), yielding 14.7 million raw paired-end reads. Adapter and quality trimming (performed with fastp 0.23.0: -q 15 -u 40 -n 5 -e 0 l 35 -w 3) (4) was followed by *de novo* assembly with SPAdes v3.5.0 (--careful --k 33,55,77,99) (5), and the assembly was polished with PrInSeS-G v.1.0.0 (6). *De novo* repeat libraries were constructed with RepeatModeler v.2.0.6 and masked via RepeatMasker v.4.1.5 (7, 8). Genome annotation (CDSs, tRNAs, rRNAs, ncRNAs, pseudogenes) was performed with National Center for Biotechnology Information PGAP v6.10 and Prokka v.1.10 (9, 10). Functional classification of predicted proteins was carried out using the Clusters of Orthologous Genes (11) and Kyoto Encyclopedia of Genes and Genomes databases (12).

The draft genome of *E. gallinarum* TY-1 comprises 3,363,870 bp assembled into 52 contigs (N50 = 208.6 kb; G + C content = 40.5%). Annotation identified 3,293 genes: 3,150 CDSs, 7 rRNAs, 56 tRNAs, four ncRNAs, and 76 pseudogenes (Table 1). Mean sequencing depth was 346.2×, and CheckM v1.2.3 estimated completeness at 92.47% with 4.8% contamination (13).

## Data Availability

Whole-genome shotgun sequence of *Enterococcus gallinarum* strain TY-1 was deposited at DDBJ/ENA/GenBank under accession JBODOG000000000 (BioProject PRJNA1266649, BioSample SAMN48682517, SRA SRR34082452).
